# What of Bismuth?

**Published:** 1884-03

**Authors:** H. V. M. Miller

**Affiliations:** Professor Practice Medicine in the Atlanta Medical College, Atlanta, Ga.


### WHAT OF BISMUTH?

BY H. V. M. MILLER, M.D ,

*Professor Practice Medicine in the Atlanta Medical College, Atlanta, Ga.*

Several salts of bismuth have been used or recommended as medicinal agents; that best known and most frequently employed is the subnitrate of bismuth.

Under the name of the magistery of bismuth, its virtues were first announced to the world by Dr. Odier, of Geneva, in 1786.

Twenty years after. it was plausibly introduced to English-speaking physicians in a paper read before the Medical Society of London by Dr. Marcett, at that time one of the physicians in Guy Hospital, who had learned its use from Dr. Odier.

With such commendation, accompanied with the assurance that it was perfectly harmless, its use extended widely; and though many physicians gave it with doubt of its efficiency, in a few years it became a common and recognized remedy. Unfortunate results occurring in a number of cases in which it was employed converted doubt into apprehension, and it began to be regarded, and was classified by Orfila, as an irritant poison.

Some of the cases, in which injury or death seemed to have been occasioned by it, became subjects of judicial investigation. In one of them, recorded in works on legal medicine, a dose of three drachms caused the death of an adult in nine days. The symptoms were burning pain in the throat, with vomiting and purging, coldness of the surface, and spasms of the arms and legs—also a strong metallic taste in the mouth. On inspection, the throat, larynx and gullet were found inflamed, and there was inflammatory redness in the stomach and throughout the intestinal canal.

In another case, by mistake six drachms of the subnitrate were taken by a man in divided doses in three days. He suffered long and distressingly from vomiting, pain in the abdomen and throat, but finally recovered.

Symptoms of poisoning of great severity occasionally following its administration challenged more careful investigation, and chemistry soon detected the presence of more or less of arsenic in nearly all the specimens of the subnitrate of bismuth then vended.

Improved processes of manufacture were soon adopted, which perfectly separated from the crude bismuth the trace of arsenic always combined with it, and guaranteed to the world the chemically pure drug as we now have it.

Thus prepared, the subnitrate of bismuth is a heavy, impalpable powder, of a pure white color, odorless, tasteless and insoluble. Introduced into the intestinal canal, it produces no sensible effect whatever. It is neither emetic nor cathartic; it neither excites nor retards peristatic or any other muscular action. It neither increases, diminishes nor alters the secretion of any gland or emunctory of the body. The most delicate chemical tests fail to detect evidence of its presence in the blood, in the urine or in any secretion of the body. The most careful observation has detected no impression whatever upon the nervous system, or the slightest change in its delicate phenomena. Whatever amount of it is introduced into the stomach passes from the body without diminution or change, except by its discolorization, probably by the sulphuretted hydrogen of the bowels. These negative facts are not invalidated by any variation in the mode of administration or the amount of the dose; it may be safely given in any quantity, from a quarter of a grain to a quarter of a pound. Free from impurities as we now have it, a poisonous dose is as unknown as if it were absolutely inert.

It is a generally accepted maxim that all medicines, when they are exhibited internally, must be dissolved in the gastro-intestinal humors before they exert any general action on the animal economy. This opinion is crystallized in the old phrase, *nihil agunt nisi soluta.* Mialhe emphasizes it in regard to chemical substances by proclaiming that "no metal, no oxide, no saline compound, can become active until it is rendered soluble.

It is difficult, therefore, to give any reasonable explanation of its mode of action. or indeed positive proof that it has any, and hence many physicians reject it, or employ it with doubt and hesitation. Still it has grown into very general use in the diseases to which it is thought to be applicable. Most frequently it is given with very indefinite notions of what it is capable of effecting, with great variation in amount, and of course with very great uncertainty of results; but amid the conflicting opinions respecting it, there is ample evidence that a large number of the profession believe that it has real and great efficiency in some forms of gastric and intestinal disorder.

A long list might be made of writers on medicine who commend it more or less strongly. "In cramps or violent pain in the stomach, and other nervous ailments of pregnant women, or any form of gastric irritability." "In functional disorders or pain in the stomach, especially when it is very irritable." "In pain in the stomach, with increased secretion of gastric acid. In infants, when this kind of disorder, with exhausting diarrhœa, results from the irritation of teething, from improper food or from a change of food in weaning." "In habitually laborious indigestion, in acid eructation of flatus, in cardialgia gastrodinia and pyrosis." "In diarrhœas, idiopathic or symptomatic, preferably in those which follow febrile attacks, which result from bad food, slow and painful digestion, or any other irritation." "In cholera, sporadic or epidemic." "In the diarrhœa following typhoid fever or phthisis pulmonalis." "Especially is it suitable for weakly children during dentition, who have diarrhœa on the slightest occasion." "In dysentery, whether rheumatismal paludal or infectious, it is wonderfully successful." "In intestinal hæmorrhage, supervening in the second period of typhoid fever, great reliance may be placed upon it."

After a century of experience with nitrate of bismuth, during nearly half of which it has been an officinal preparation, no agreement has been reached as to the proper dose in which it should be administered in order to secure the good results to be expected from it. By different persons it has been given without limitation as to age, in amount varying from a few grains three times a day to a teaspoonful or a tablespoonful given every hour in the day. No sensible effects follow its administration; but hundreds of observers assert that in a long list of ailments prompt cures result from its use. Unwilling to deny the correctness of this observation, professional ingenuity has suggested several explanations of them more or less plausible, if true. In view of the obvious symptoms and ascertained pathology of most of the diseases which nitrate of bismuth is claimed to mitigate or cure, a rational physician would be likely to prescribe one of the several classes of remedies known as antispasmodics, sedatives, astringents, antacids or absorbents. To lessen the labor of investigation, it is asserted that this drug possesses the properties and powers of one or all of these classes of the materia medica. This assertion may well be questioned. Antispasmodics allay abnormal muscular action, whether it occur in the voluntary or involuntary system; this action can only be effected by impressing the system of nerves, which respectively preside over the muscles. Sedatives exert an influence over the nervous system, by which its energy is weakened or suspended, sensibility lessened, pain arrested, and preternatural muscular contraction (spasm), always dependent on nervous energy, terminated.

This influence can never be produced by articles which are insoluble, and which can in no way make a direct impression on the nervous filaments, or indirectly upon the ganglia after absorption, through the medium of the circulation.

Astringents gain entrance to the system through the circulation, after solution and absorption, and also produce their effect by an impression upon the nervous system, but in a different direction they excite the contractility of the capillary vessels, lessen their caliber and limit the normal and abnormal exhalation from them. Locally both these classes of articles exhibit their effects unmistakably in a degree proportioned to the dose.

Antacids are simply alkaline substances, capable of neutralizing acids present in the intestinal canal or circulating in the blood. Their action depends upon their perfect solubility and ready chemical decomposition and quick absorption, none of which properties are possessed by the nitrate of bismuth.

Absorbents are articles which imbibe a limited amount of fluid in which they may be immersed, as dry lint or sponge will do; any dry powder will become wet with any fluid; dry nitrate of bismuth can do no more. Indeed, it is generally prevented from doing this much, as it is usually given in water, gruel or some other fluid.

Possessing clearly none of the well known and easily recognized properties of these classes of medicines, to what are its curative powers to be attributed? The solitary attempt to answer this question has been fairly made by M. Moueret, a French physician, who was the pioneer in the administration of it in the large doses now become common. After years of experiments with it, he commends it in the strongest terms in all the diseases enumerated above, and in others of cognate character. The following sentences on the administration and action of the remedy, though evincing enthusiasm, present his views clearly and are copied entire:

"*Administration and action.*—It should be given in powder, and best so with the first spoonful of broth or gruel. It excites no disgust, especially if placed between two bits of bread soaked in broth. Children take it readily with milk or ptisans. The author has never given less than from two to three drachms per diem, nor more than twenty, and he has never observed the slightest inconvenience from these large doses; and it is his custom to give it to the children in his hospital by spoonfuls, or table-spoonfuls, without observing more exactitude, so innocuous is it. So imperfectly is this fact known, that the chemists hesitate in preparing his prescriptions in which these large doses are ordered. The author cannot conceive why this substance was ever set down as an irritant poison, as he has never found the slightest irritation from the largest doses given to either the healthy or the sick; and post-mortem examination proves that, beyond patches of black discoloration, it produces no effect on the mucous membrane, the consistence of this remaining quite normal. The action of the substanceon the canal seems to be quite negative—that is to say, the abnormal symptoms for which it was prescribed quickly disappear. Great attention has failed also to detect any marked effect upon any other part of the system. Perhaps the urine is somewhat increased, and the pulse becomes slower, but this is probably due to the relief of the gasto-intestinal affection. The action of bismuth is then purely a *local* one ; and this local action, so far from being an irritant one, as so commonly stated, diminishes the activity of the phenomena of which the mucous membrane is the seat. It is not to be supposed, however, that this inert substance can act actively and directly upon the canal, as a soluble body after absorption does do ; and it is probable that its acts merely as an inert body, just as charcoal might do, mechanically protecting the over-excited secretory organs and papillæ of the nerves from the too immediate contact of the fluids, and especially of aliments. By its mechanical apposition, it limits the amount of exhalation. The allegation of its antispasmodic properties has solely risen from observing the diminution of the symptoms, without considering whether this might not arise negatively from the preservation of the gastro-intestinal surface from the causes of irritation. In this way we might call darkness an antispasmodic, because it allows the repose of the retina, and the subdual of the irritation which gives rise to photophobia.—*Gazette Medicale*, Nos. 15, 16."

This explanation is not only mechanical, but purely hypothetical, and by no means satisfactory to the rational inquirer. But as medicine, in its most important branch, therapeutics, is much less indebted to rationalism than to observation and experience, the use of nitrate of bismuth may be justified as a purely empyrical remedy, if clearly proven facts demonstrate its value. When no relation can be traced between the giving of a remedy and the disappearance of certain symptoms, the mere fact is of little value as a guide to practice; the weakness of such observations, unconnected by reason, can only be overcome by the force of numbers. Great numbers of like cases are not easily got together; thirty or forty observations may establish the diagnosis and prognosis of a disease, but many years of researches and thousands of cases are required to reach a satisfactory conclusion in empyrical therapeutics.

The lists of cures said to be effected by thousands of remedies, new and old, published from time to time by empyrics, in and out of the profession, serve to show the danger of accepting too trustingly and implicitly unverified opinions for ascertained facts.

Several circumstances connected with the medical history of nitrate of bismuth are calculated to throw more or less of doubt on some of the published opinions of its power to cure disease.

In the first place, the diseases or symptoms, for the relief of which it is given, are without exception those in which spontaneous cures, or occasional pauses in their progress, are of most frequent occurrence, and in which, even when combatted by drugs of known powers, producing sensible effects, it is often very difficult honestly to determine the remedial action of the medicines administered.

Secondly. In a large majority of instances it is given in combination with other medicines having more or less power over the morbid conditions or symptoms present: with opium, magnesia, lime water, soda, camphor, assafœtida; with kino, catechu, krameria, logwood, tanin; with any of the vegetable astringents; with lead, iron or ergot; with any of the substances known independently of it, to have the effect of allaying pain, relaxing spasm, neutralizing excess of acid, restraining undue secretion from the stomach or bowels. When combined with any of these drugs, how is it possible to form any well-grounded opinions of the value or individual effect of each?

As an example of the frequent manner of combining bismuth with other articles, take this account of a case of idiopathic dysentery in the Royal Free Hospital (London), of a young man who on his admission was having as many as twenty dysenteric motions per diem: "Under the influence of a mixture, consisting of a scruple of bismuth, ten grains compound powder of kino, two drachms of mucilage, and an ounce of infusion of krameria, every six hours, conjoined with enemata of forty drops of tincture of opium, two drachms of tincture of catechu and two ounces of decoction of starch, every night. The stools diminished to one daily within a fortnight; at the same time the most careful attention was paid to his diet. The treatment pursued here proved highly satisfactory, and is well worthy of extended trial in dysenteric complaints, and is especially recommended in the diarrhœa of phthisis, and also that of typhoid fever and the chronic diarrhœa of children." The Doctor cautiously adds that he "cannot say that the good effects were solely due to the bismuth."

Thirdly. When given uncombined the irregularity of the dose and varying frequency of its administration in all cases, without the production of marked results in any, precludes the possibility of correctly estimating its powers or of proving that it possesses any. To adults, in colliquative diarrhœas, idiopathic or consequitive of intestinal lesion, it is highly commended in doses of five grains every six hours; to children, in cholera infantum, it is confidently advised in teaspoonful doses every hour. In the absence of all knowledge of its *modus operandi*, *caprice* or *impulse* appear to be the sole guide to the amount to be given or to the proper mode of its administration.

Long ago dry dirt, or to adopt more elegant language, various forms of argillaceous earth, purified by trituration and elutriation from certain native earths existing in different countries and known as Arminian Bole, was employed most extensively and enjoyed high repute in the very same conditions in which the nitrate of bismuth is now recommended. Identically the same properties were claimed for it; it was said to be absorbent, antacic, antispasmodic and astringent. Its use was justified by what is called high authority. Dispensatories lauded it, book-writers approved it, general professional approbation sanctioned it, opinion of its valuable curative power was well-nigh universal. But opinion is not evidence, and closer scrutiny of its supposed effects led to its rejection as inert, and to its relegation to the humble office of a dentifrice.

This is by no means a solitary instance in which philosophical investigation and analysis of alleged facts have eliminated from plethoric dispensatories and from medical literature worthless or hurtful drugs. This process ought to be continued; this field of useful labor is large and inviting. It is not improbable that in the near future, under the same methods pursued, hundreds of articles long enumerated and tediously continued in works on materia medica will be shown to merit like exclusion. Whether nitrate of bismuth is one of these is yet to be determined. Its reputation rests mainly upon the impression made upon the minds of isolated observers, who have employed it in spontaneously curable diseases, and often combined with articles of well known efficacy. Doubtless it has been given uncombined with other drugs, and thus one source of error avoided, but the number of such cases and the exact condition of each have not been stated with such clearness as to aid the judgment. No attempt has been made to furnish statistics in a manner and in sufficient number to demonstrate its great therapeutical value; something more is needed than the statement of the fact that patients have recovered after its administration. It should be shown that more recoveries occur by means of its use than by other well understood methods of treatment, or that the duration of the disease is shortened.

New and more careful study of its effects is demanded, in which all possible sources of error or misinterpretation shall be avoided, the exact facts in regard to its therapeutical properties ascertained, and a rational explanation of those furnished if it long retain and justify its present reputation.

Whatever may be now or hereafter the professional opinion of the nitrate of bismuth, as a medicine, it is probable that under the name of pearl white it will hold its place as a cosmetic. Its value in this respect is admitted and has been celebrated in lofty poetic numbers by Alexander Pope:

> "First, robed in white, the nymph intent adores,
> With head uncovered, its cosmetic powers."

